# Role of miR-34a-5p in Hematopoietic Progenitor Cells Proliferation and Fate Decision: Novel Insights into the Pathogenesis of Primary Myelofibrosis

**DOI:** 10.3390/ijms18010145

**Published:** 2017-01-13

**Authors:** Elisa Bianchi, Samantha Ruberti, Sebastiano Rontauroli, Paola Guglielmelli, Simona Salati, Chiara Rossi, Roberta Zini, Enrico Tagliafico, Alessandro Maria Vannucchi, Rossella Manfredini

**Affiliations:** 1 Centre for Regenerative Medicine “Stefano Ferrari“, Department of Life Sciences, University of Modena and Reggio Emilia, 41125 Modena, Italy; samantha.ruberti@unimore.it (S.R.); sebastiano.rontauroli@unimore.it (S.R.); simona.salati@unimore.it (S.S.); chiara.rossi@unimore.it (C.R.); roberta.zini@unimore.it (R.Z.); 2 CRIMM, Center for Research and Innovation for Myeloproliferative Neoplasms, AOU Careggi and Department of Experimental and Clinical Medicine, University of Florence, 50134 Florence, Italy; paola.guglielmelli@unifi.it (P.G.); a.vannucchi@unifi.it (A.M.V.); 3 Center for Genome Research, University of Modena and Reggio Emilia, 41125 Modena, Italy; enrico.tagliafico@unimore.it

**Keywords:** miR-34a-5p, nuclear receptor subfamily 4, group A, member 2 (NR4A2), lymphoid enhancer-binding factor 1 (LEF1), MYB, hematopoetic progenitor cells, hematopoietic differentiation, megakaryopoiesis, primary myelofibrosis, myeloproliferative neoplasms, macrophage

## Abstract

Primary Myelofibrosis (PMF) is a chronic Philadelphia-negative myeloproliferative neoplasm characterized by a skewed megakaryopoiesis and an overproduction of proinflammatory and profibrotic mediators that lead to the development of bone marrow (BM) fibrosis. Since we recently uncovered the upregulation of miR-34a-5p in PMF CD34+ hematopoietic progenitor cells (HPCs), in order to elucidate its role in PMF pathogenesis here we unravelled the effects of miR-34a-5p overexpression in HPCs. We showed that enforced expression of miR-34a-5p partially constrains proliferation and favours the megakaryocyte and monocyte/macrophage commitment of HPCs. Interestingly, we identified lymphoid enhancer-binding factor 1 (LEF1) and nuclear receptor subfamily 4, group A, member 2 (NR4A2) transcripts as miR-34a-5p-targets downregulated after miR-34a-5p overexpression in HPCs as well as in PMF CD34+ cells. Remarkably, the knockdown of NR4A2 in HPCs mimicked the antiproliferative effects of miR-34a-5p overexpression, while the silencing of LEF1 phenocopied the effects of miR-34a-5p overexpression on HPCs lineage choice, by favouring the megakaryocyte and monocyte/macrophage commitment. Collectively our data unravel the role of miR-34a-5p in HPCs fate decision and suggest that the increased expression of miR-34a-5p in PMF HPCs could be important for the skewing of megakaryopoiesis and the production of monocytes, that are key players in BM fibrosis in PMF patients.

## 1. Introduction

Primary myelofibrosis (PMF) is a Philadelphia-negative (Ph^−^) myeloproliferative neoplasm (MPN). MPNs are a group of clonal hematological malignancies that include polycythemia vera (PV) and essential thrombocythemia (ET), together with PMF [1]. The mutation of a single hematopoietic stem cell (HSC) underlies the clonal expansion and the myeloproliferative phenotype that all the MPNs share [2].

The abnormal expansion of megakaryopoiesis with maturation defects and the bone marrow (BM) fibrosis are the main features of PMF. Indeed, the presence of hyperplastic and dysplastic megakaryocytes and the detection of fibrosis in the BM are the main criteria for differential diagnosis of PMF from ET and PV, according to the World Health Organization (WHO) classification of Ph^−^ MPNs [1,3].

The aberrantly differentiated megakaryocytes which accumulate in the BM play a major role in the release of proinflammatory and profibrotic cytokines, chemokines and growth factors that in turn drive the development of BM fibrosis, osteosclerosis and eventually extramedullary hematopoiesis in PMF patients. Together with megakaryocytes, monocytes are involved in the overproduction of proinflammatory and profibrotic mediators that fuel the development of fibrosis and other alterations of the BM architecture in PMF patients [4,5,6,7].

MPNs mostly result from genomic lesions in janus kinase 2 (*JAK2*), thrombopoietin receptor (*MPL*) and calreticulin (*CALR*) genes, also known as “MPN driver mutations”, that account for the abnormal activation of JAK2 signaling pathway and, ultimately, for the clonal proliferation [8,9,10,11]. Despite the efforts to elucidate the genomic landscape of PMF, none of the genomic lesions identified till now is able to completely explain all the pathological features of PMF. Therefore, further efforts should be directed to elucidating the molecular mechanisms behind the skewed crosstalk between the malignant clone-derived hematopoietic cells and BM microenvironment. This is of paramount importance to identifying novel therapeutic targets to restrain the abnormal megakaryopoiesis and to constrain the overwhelming production of proinflammatory and profibrotic mediators that underlie the onset of the BM fibrosis and osteosclerosis.

To address this issue, we have recently compared the gene and miRNA expression profiles of CD34+ hematopoietic progenitor cells (HPCs) from PMF patients and healthy donors (HDs) [12]. This study uncovered the deregulated expression of many genes and miRNAs in PMF HPCs potentially involved in PMF pathogenesis. Notably, microarray data unveiled the upregulation of miR-34a-5p in CD34+ HPCs from PMF patients. Since miR-34a-5p was reported to enhance the PMA-induced megakaryocytic differentiation of K562 cell line [13], we wondered whether and how the upregulation of miR-34a-5p in PMF HPCs could have a role in PMF pathogenesis.

Here we showed that the enforced expression of miR-34a-5p in CD34+ HPCs negatively interferes with cell proliferation and favours the commitment toward the megakaryocyte and macrophage lineages. Furthermore, we demonstrated that the effects of miR-34a-5p overexpression on the HPCs proliferation and fate decision are mediated by the downregulation of its targets lymphoid enhancer-binding factor 1 (LEF1) and nuclear receptor subfamily 4, group A, member 2 (NR4A2).

## 2. Results

### 2.1. miR-34a-5p Expression Levels in CD34+ Cells from PMF Patients and HDs

Microarray data of human HPCs from 38 PMF (*n* = 20 *JAK2*-mutated, *n* = 3 *MPL*-mutated, *n* = 9 *CALR*-mutated and *n* = 6 triple-negative) and 30 HDs were collected from a dataset that we previously published [12] and deposited in the GEO repository (http://www.ncbi.nlm.nih.gov/geo; series GSE53482).

**Figure 1:**
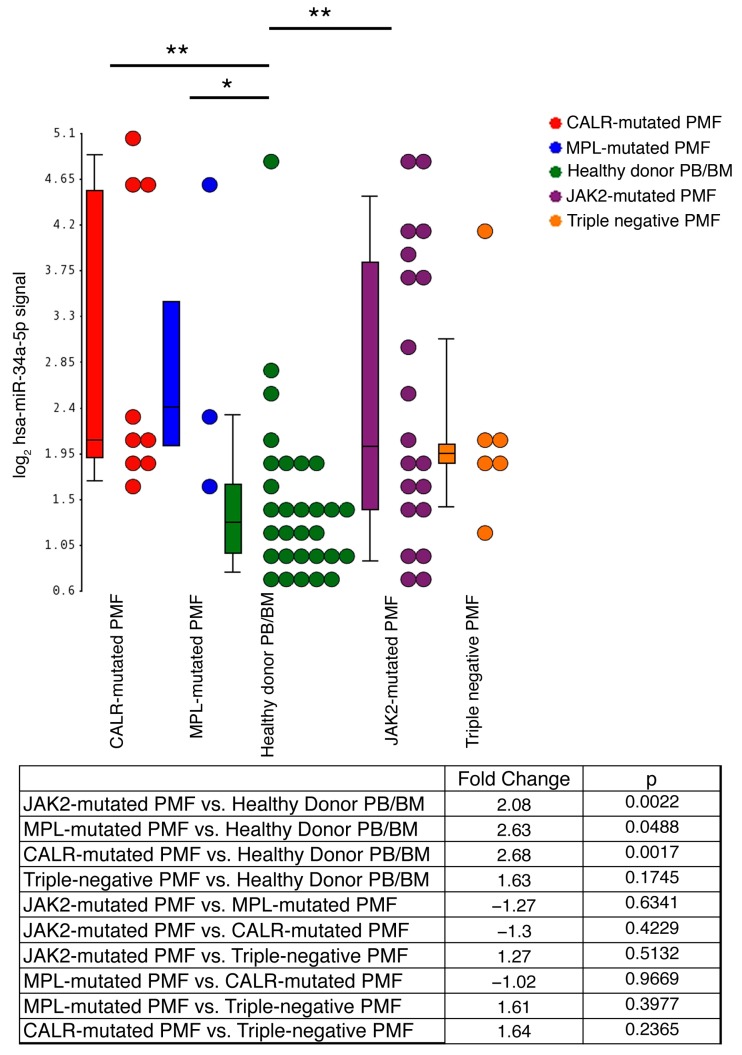
Expression levels of miR-34a-5p in CD34+ cells from primary myelofibrosis (PMF) patients and healthy donors (HDs). Gene expression levels were measured by microarray analysis performed by Affymetrix platform as detailed in Materials and Methods Section 4.2. miR-34a-5p expression levels are reported as Robust Multiarray Analysis (RMA)-normalized log2 signals, which were obtained by using the Partek^®^ Genomics Suite^®^ software, version 6.6 Copyright^©^, 2016 (Partek Inc., St. Louis, MO, USA; http://www.partek.com). Boxes represent the interquartile range that contains 50% of the subjects, the horizontal line in the box marks the median, and bars show the range of values. Data are representative of 38 PMF and 30 HD samples (14 from bone marrow and 16 from peripheral blood). PMF samples are classified into *JAK2*-mutated (*n* = 20), *MPL*-mutated (*n* = 3), *CALR*-mutated (*n* = 9) and triple-negative (*n* = 6), based on the mutational status. The table at the bottom of the figure displays the results (Fold Change and *p*-value) for the comparative analysis of *JAK2*-mutated, *MPL*-mutated, *CALR*-mutated and triple negative PMF subgroups with the healthy donor-derived samples. Abbreviations: PMF, primary myelofibrosis; PB, peripheral blood, BM, bone marrow; *n* = number of samples. * *p* < 0.05; ** *p* < 0.01.

As previously reported, miR-34a-5p level was remarkably higher in PMF patients compared with HDs (Fold Change (FC) = 2.16, FDR = 4.75 × 10^−3^; Figure 1). Notably, the comparative analysis of each subgroup of PMF patients based on their mutational status with the HD samples unveiled a remarkable increase of miR-34a-5p expression levels in *JAK2*-mutated, *MPL*-mutated and *CALR*-mutated vs. HDs (FC ≥ 2, *p* < 0.05; Figure 1), while this trend was weaker and not statistically significant for triple-negative PMF samples vs. HDs (FC = 1.628; *p* = 0.17; Figure 1). In addition, no remarkable difference in miR-34a-5p expression levels was detected thorugh the pairwise comparison among the *JAK2*-mutated, *MPL*-mutated and *CALR*-mutated PMF subsets (Figure 1).

### 2.2. Effects of miR-34a-5p Overexpression on HPCs Proliferation and Clonogenic Efficiency

In order to unravel whether miR-34a-5p could be relevant for HPCs proliferation and lineage choice, we studied the effects of its overexpression in healthy donor cord blood (CB)-derived CD34+ cells by means of miR-34a-5p mimic transfection (miR-34a-5p mimic), compared with a negative control mimic-transfected sample (NegCTR mimic). A set of three independent experiments starting from different HD-derived CB units was performed. The effective upregulation of miR-34a-5p upon miR-34a-5p mimic transfection was checked 24 h after the last nucleofection (hereafter reported as *post-nucleofection*) by Reverse Transcription Quantitative Polymerase Chain Reaction (RT-qPCR) (RQ = 470.3 ± 224.7, mean ± SD, in miR-34a-5p mimic vs. NegCTR mimic, *p* = 0.034).

**Figure 2:**
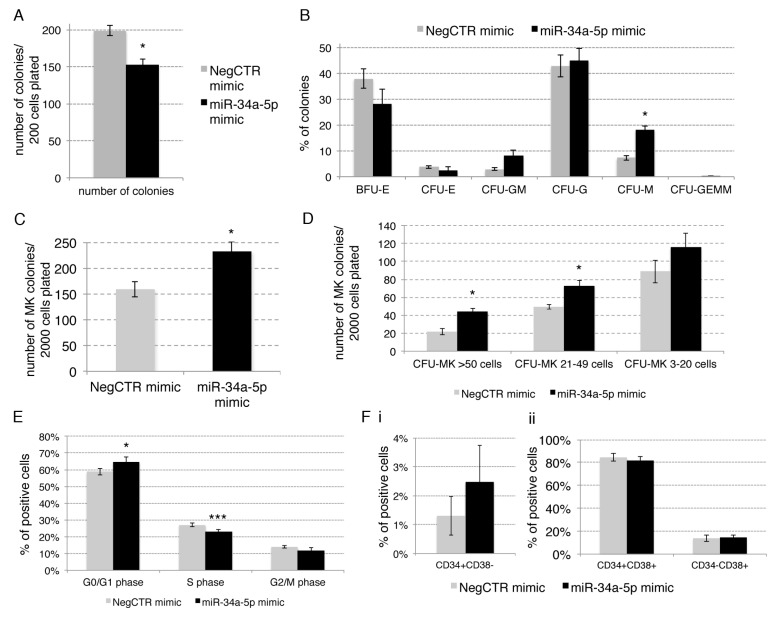
Effects of miR-34a-5p overexpression on CD34+ hematopoietic progenitor cells clonogenic activity, proliferation and commitment. (**A**,**B**) Methylcellulose clonogenic assay results (mean ± SEM; *n* = 3). The colony scoring results are reported as total number of colonies grown from 200 cells plated (**A**) and percentages of each colony type (**B**); Colonies were scored according to the manufacturer’s guidelines; (**C**,**D**) Megakaryocyte clonogenic assay results (mean ± SEM; *n* = 3) in terms of megakaryocyte colony number (**C**) and size (**D**); Values are reported as number of megakaryocyte colonies for 2000 plated cells; (**E**) Statistical analysis results (mean ± SEM; *n* = 3) for the percentage of cells in the different cell cycle phases performed by propidium iodide staining 24 h post-nucleofection; (**F**) Flow cytometric detection (mean ± SEM; *n* = 3) of the CD34+CD38− hematopoetic stem cell fraction (**Fi**) and the CD34+CD38+ and CD34−CD38+ hematopoietic progenitor cell populations (**Fii**) at 24 h post-nucleofection. Data are from *n* = 3 independent experiments performed with different healthy donor-derived cord blood units. Error bars in the graphs represent SEM. * *p* ≤ 0.05 and *** *p* ≤ 0.001 vs. NegCTR mimic sample. Abbreviations: BFU-E, burst forming unit-erythroid; CFU-E, colony forming unit-erythroid; CFU-GM, colony forming unit-granulocyte/monocyte; CFU-G, colony forming unit-granulocyte; CFU-M, colony forming unit-monocyte; CFU-GEMM, colony forming unit-granulocyte/erythroid/monocyte/megakaryocyte; MK, megakaryocyte; CFU-MK, colony forming unit-megakaryocyte; *n* = number of experiments.

To investigate the role of miR-34a-5p in HPCs fate decision, we firstly evaluated the effects of miR-34a-5p overexpression on the HPCs clonogenic activity by methylcellulose and collagen-based clonogenic assays. Methylcellulose assay highlighted a reduction of the clonogenic efficiency of miR-34a-5p mimic CD34+ cells compared with the NegCTR mimic sample (Figure 2A).

Furthermore, the overexpression of miR-34a-5p induced a remarkable increase of the percentage of monocyte colony forming units (CFU-M), while the erythroid (burst-forming unit-erythroid, BFU-E and CFU-E), the granulocyte (CFU-G) and the granulocyte/monocyte (CFU-GM) colonies were not significantly affected (Figure 2B).

We also examined the effect of miR-34a-5p overexpression on the megakaryocyte commitment by plating NegCTR mimic and miR-34a-5p mimic CD34+ cells in a collagen-based serum-free semisolid culture medium which supports the growth of megakaryocyte progenitors in vitro. The results, reported in Figure 2C,D, showed that miR-34a-5p overexpression in CD34+ cells strongly increased the number of megakaryocyte colony forming units (CFU-MKs; Figure 2C). In addition, CFU-MKs were scored based on their size, which reflects the maturation stage of the progenitors they originate from. CFU-MK scoring showed that medium (21–49 cells) and large (>50 cells) CFU-MKs accounted for the most remarkable differences between miR-34a-5p mimic and NegCTR mimic samples (Figure 1D). Since large and medium CFU-MKs come from more primitive megakaryocyte progenitors whereas small CFU-MKs originate from more mature megakaryocyte progenitors, these data strongly suggest that miR-34a-5p overexpression affects the earliest phases of megakaryopoiesis.

To understand how miR-34a-5p could affect the HPCs clonogenic ability (Figure 2A), we analysed the effects of miR-34a-5p on CD34+ cells proliferation by Propidium Iodide (PI) staining and flow cytometric analysis of cell cycle distribution. Cell cycle analysis at 24 h post-nucleofection revealed that miR-34a-5p overexpression slightly but significantly induced a delay in G1-to-S phase progression, as demonstrated by the increase of the G1-phase cell fraction and the decrease of the S-phase fraction in comparison with the NegCTR mimic sample (Figure 2E and Figure S1A). Furthermore, we evaluated the HPCs commitment by flow cytometric analysis of CD34 and CD38 markers at 24 h post-nucleofection. Flow cytometry data showed that no significant difference could be detected between miR-34a-5p mimic and NegCTR mimic samples as far as the CD34+CD38− fraction, which is enriched in HSC and uncommitted progenitors (Figure 2Fi and Figure S1B), and the CD34+CD38+ population, that is endowed of a more restricted lineage commitment potential [14], are concerned (Figure 2Fii and Figure S1B).

Collectively, these results indicated that the overexpression of miR-34a-5p in CD34+ HPCs partially constrains G1-to-S phase cell cycle progression that in turn leads to a reduction of their clonogenic efficiency. Moreover, our data suggested that miR-34a-5p affects the macrophage commitment together with the megakaryocyte one.

### 2.3. Effects of miR-34a-5p Overexpression on HPCs Lineage Choice

**Figure 3:**
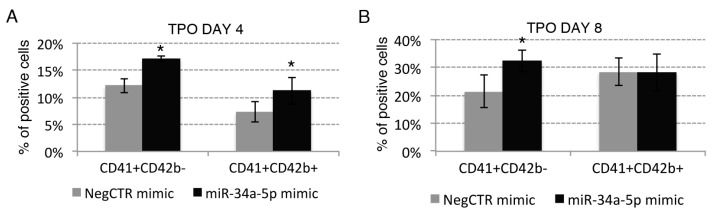

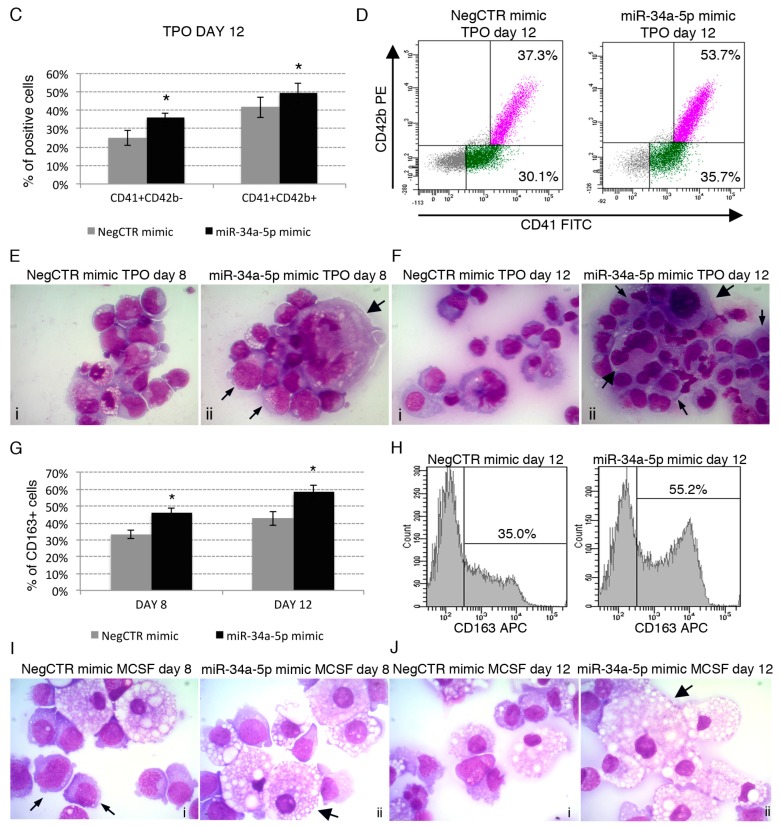
Effects of miR-34a-5p overexpression on megakaryocyte and macrophage differentiation. (**A**–**C**) Flow cytometric analysis (mean ± SEM; *n* = 3) of CD41 and CD42b expression at day 4 (**A**); day 8 (**B**) and day 12 (**C**) of TPO-driven megakaryocyte unilineage culture post-nucleofection; (**D**) Representative dot plots for the flow cytometric detection of CD41 and CD42b differentiation markers at day 12 of megakaryocyte unilineage culture post-nucleofection; (**E**,**F**) Morphological analysis of NegCTR mimic (**i**) and miR-34a-5p mimic-transfected cells (**ii**) after May–Grunwald–Giemsa staining at day 8 (**E**) and day 12 (**F**) of megakaryocyte unilineage culture post-nucleofection in a representative experiment. Thick arrows indicate large megakaryocytes with polyploid nuclei; thin arrows indicate cells with a granular cytoplasm, which is a feature of megakaryocyte maturation. Magnification, ×1000; (**G**) Flow cytometric detection (mean ± SEM; *n* = 3) of the CD163 marker at day 8 and day 12 of monocyte/macrophage unilineage culture post-nucleofection; (**H**) Representative histograms for the flow cytometric detection of CD163 marker at day 12 of MCSF-driven monocyte/macrophage unilineage culture post-nucleofection; (**I**,**J**) Morphological analysis of NegCTR mimic (**i**) and miR-34a-5p mimic-transfected cells (**ii**) after May–Grunwald–Giemsa staining at day 8 (**I**) and day 12 (**J**) of monocyte/macrophage unilineage culture post-nucleofection in a representative experiment. Thick arrows indicate macrophages; thin arrows indicate monocytes. Magnification, ×1000. Three independent experiments (indicated by *n* = 3) were performed with different healthy donor-derived cord blood units. * *p* ≤ 0.05 in miR-34a-5p mimic compared to NegCTR mimic sample. Abbreviations: TPO, thrombopoietin; MCSF, monocyte colony-stimulating factor.

To shed some light into the role of miR-34a-5p in HPCs fate decision, we further investigated the effects of miR-34a-5p overexpression on CD34+ cells commitment by flow cytometric analysis of differentiation markers and morphological analysis of May-Grunwald-Giemsa (MGG)-stained cytospins in unilineage megakaryocyte, monocyte/macrophage, erythroid and granulocyte culture conditions (Figure 3 and Figure S2).

Our data further demonstrated that miR-34a-5p overexpression enhances the TPO-induced megakaryocyte differentiation. Indeed, flow cytometry data highlighted an increase of the megakaryocyte-committed population from the earlier CD41+CD42b− to the more mature CD41+CD42b+ fraction in miR34a-5p mimic compared to NegCTR mimic cells at 4, 8 and 12 days of TPO-driven megakaryocyte culture (Figure 3A–D). Consistently, morphological analysis at 8 and 12 days of culture (Figure 3E,F) showed that miR-34a-5p mimic samples were enriched in large megakaryocytes with polyploid nuclei that result from endomitosis and mature megakaryocytes with a highly complex and granular cytoplasm (Figure 3Eii,Fii), that is a feature of megakaryocyte maturation, compared to NegCTR mimic samples (Figure 3Ei,Fi).

Moreover, miR-34a-5p overexpression enhanced the MCSF-driven macrophage differentiation, as demostrated by the increase of the population positive for the macrophage-specific marker CD163 in miR-34a-5p mimic compared to NegCTR mimic sample at 8 and 12 days of culture (Figure 3G,H).

Indeed, the monocyte/macrophage population induced by lineage-specific culture conditions displayed a remarkable enrichment in macrophages in miR-34a-5p-overexpressing samples in comparison with controls, as demonstrated by the morphological analysis of MGG-stained cytospins at 8 and 12 days of culture (Figure 3I,J).

We similarly studied the effects of miR-34a-5p overexpression on CD34+ cells erythroid and granulocyte differentiation but we failed to detect any significant modulation (Figure S2) Indeed, the flow cytometric analysis of the early CD36+GPA−, intermediate CD36+GPA+ and late CD36−GPA+ erythroid populations did not highlight any remarkable difference between miR-34a-5p mimic and NegCTR mimic samples at 4 and 8 days of EPO-induced erythroid differentiation (Figure S2A,B).

In line with these observations, both NegCTR mimic and miR-34a-5p mimic samples were similarly represented by proerythroblasts at 8 days of EPO-induced erythroid differentiation (Figure S2C).

In a similar way, flow cytometric analysis did not detect any remarkable difference in MPO-positive cells between miR-34a-5p mimic and NegCTR mimic samples during the granulocyte colony-stimulating factor (GCSF)-driven granulocyte differentiation (8 and 12 days, Figure S2D). Additionally, morphological analysis of MGG-stained cytospins demonstrated that both miR-34a-5p overexpressing cells and control were mainly represented by myelocytes and band neutrophils, without any remarkable difference (Figure S2E).

Collectively these data demonstrate that the enforced expression of miR-34a-5p in CD34+ HPCs favours their differentiation toward the megakaryocyte and macrophage lineages. In contrast, the erythroid and granulocyte lineages were not significantly affected by miR-34a-5p overexpression.

### 2.4. Gene Expression Profile (GEP) of miR-34a-5p-Overexpressing Cells and Identification of miR-34a-5p Targets

In order to shed some light on the molecular mechanisms underlying the effects of miR34a-5p in HPCs proliferation and fate decision, we investigated the transcriptional changes induced by miR-34a-5p overexpression in CD34+ cells.

For each of three experiments performed (please see Section 2.2), miR-34a-5p mimic and NegCTR mimic-transfected CD34+ cells were profiled by Affymetrix U219 array. GEP analysis disclosed 34 differentially expressed genes (DEGs) between miR-34a-5p mimic and NegCTR mimic samples (Table S1; GEP data were deposited in the GEO repository at http://www.ncbi.nlm.nih.gov/geo; series GSE89156).

Next, in order to identify the mRNAs targeted by miR-34a-5p and potentially involved in miR-34a-5p-driven control of HPCs proliferation and lineage commitment and PMF pathogenesis, we selected from the DEGs (Table S1) the transcripts downregulated upon miR-34a-5p overexpression. The resulting list was represented by 18 genes (Table 1). Among them, only the *LEF1*, *NR4A2* and *AREG1* transcripts displayed putative miR-34a-5p target sites in the 3′-UTR based on TargetScan prediction (http://www.targetscan.org), and were downregulated in PMF vs. HD CD34+ cells (Table 1). Owing to their role as transcriptional regulators, we focused our attention on LEF1 and NR4A2. Notably, both these genes have already been experimentally validated as miR-34a-5p targets by Luciferase reporter assay [15,16].

**Figure 4:**
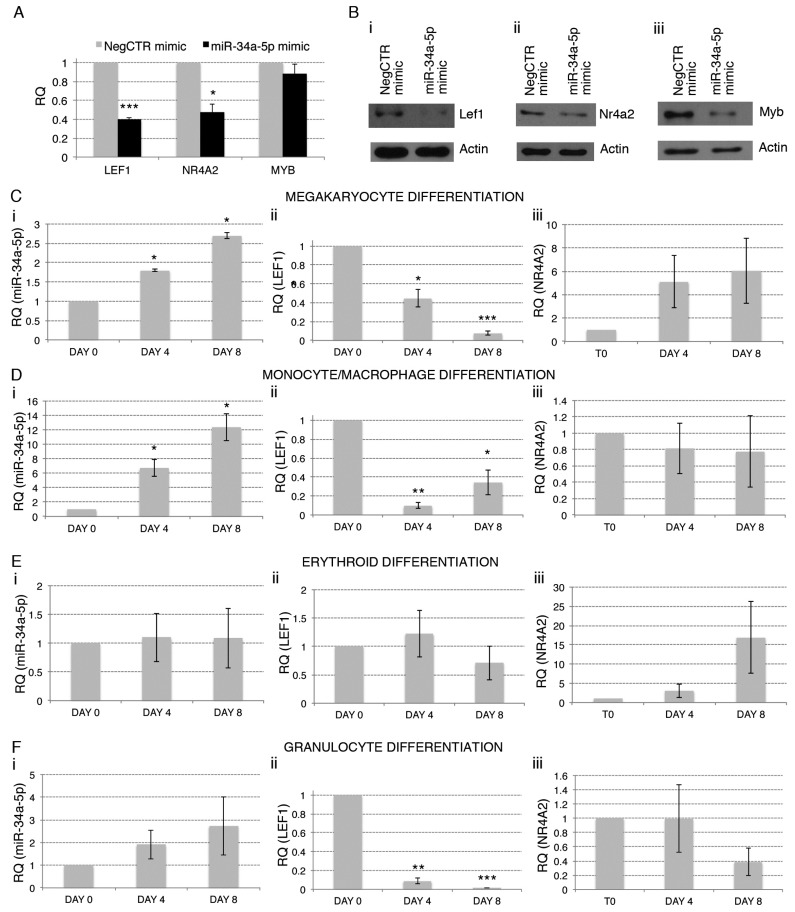
Relationship between the expression of miR-34a-5p and that of the miR-34a-5p targets during the HPCs commitment. (**A**) Detection of lymphoid enhancer-binding factor 1 (*LEF1*), nuclear receptor subfamily 4, group A, member 2 (*NR4A2*) and *MYB* expression levels by Reverse Transcription Quantitative Polymerase Chain Reaction (RT-qPCR) in Negative Control (NegCTR) mimic and miR-34a-5p mimic-transfected CD34+ cells at 24 h post-nucleofection; (**B**) Western Blotting analysis of Lef1 (**i**), Nr4a2 (**ii**) and Myb (**iii**) protein levels in protein lysates from miR-34a-5p mimic-transfected compared to NegCTR mimic-transfected CD34+ cells at 24 h after the last of two nucleofection cycles. Actin protein levels are reported as loading control; (**C**–**F**) Expression kinetics of miR34a (**i**), *LEF1* (**ii**) and *NR4A2* (**iii**) during the megakaryocyte (**C**); monocyte/macrophage (**D**); erythroid (**E**) and granulocyte (**F**) differentiation of CD34+ cells. miR-34a-5p, *LEF1* and *NR4A2* expression levels were monitored by RT-qPCR at day 4 and day 8 post-CD34+ cells purification. Modulations of miR-34a-5p, *LEF1* and *NR4A2* transcripts are reported as Relative Quantity (RQ) respect to freshly purified CD34+ cells (DAY 0) sample, which was set as calibrator. Data are from *n* = 3 independent experiments performed with different healthy donor-derived cord blood units. Values in the graph are reported as mean ± SEM. * *p* ≤ 0.05, ** *p* ≤ 0.01 and *** *p* ≤ 0.001 compared to freshly purified CD34+ cells (DAY 0) sample.

We next confirmed by RT-qPCR (Figure 4A) and Western Blot (Figure 4Bi,ii) the downregulation of *LEF1* and *NR4A2* upon miR-34a-5p overexpression. We also included MYB, which is a well-known target of miR-34a-5p [13] and a key regulator of HPCs proliferation and lineage commitment [17,18]. In line with GEP data (Table 1 and Table S2), we did not detect the downregulation of MYB transcript upon miR-34a-5p overexpression by RT-qPCR (Figure 4A). However, Western Blot highlighted a remarkable downregulation of Myb protein in miR-34a-5p mimic-transfected CD34+ cells compared with control (Figure 4Biii), by further stressing the idea that MYB acts as a key regulator of miR-34a-5p-dependent tuning of HPCs proliferation and commitment. The role of MYB in these processes has already been extensively investigated [8,17,18]. Therefore, here we focused our efforts to elucidate the role of LEF1 and NR4A2 in miR-34a-5p-mediated control of the HPCs proliferation and fate decision. For this purpose, we firstly checked by RT-qPCR the expression of miR-34a-5p, *LEF1* and *NR4A2* during the megakaryocyte, macrophage, granulocyte and erythroid differentiation of CD34+ cells. RT-qPCR data highlighted the upregulation of miR-34a-5p and the concurrent downregulation of *LEF1* during the megakaryocyte and the monocyte/macrophage differentiation (Figure 4Ci,ii,Di,ii), with no modulations were detected during the erythroid one (Figure 4Ei,ii). In addition, despite the not significant increase of miR-34a-5p expression (Figure 4Fi), *LEF1* resulted strongly downregulated during the granulocyte differentiation (Figure 4Fii). Finally, we were unable to detect an anticorrelated expression trend for miR-34a-5p and *NR4A2* during HPCs differentiation (Figure 4Ciii,Diii,Eiii,Fiii).

### 2.5. Effects of LEF1 and NR4A2 Silencing on HPCs Proliferation and Clonogenic Efficiency

To get further insights into the role of LEF1 and NR4A2 in regulating the HPCs proliferation and lineage commitment, we silenced their expression in CB-derived CD34+ HPCs through the nucleofection of siRNAs.

**Figure 5:**
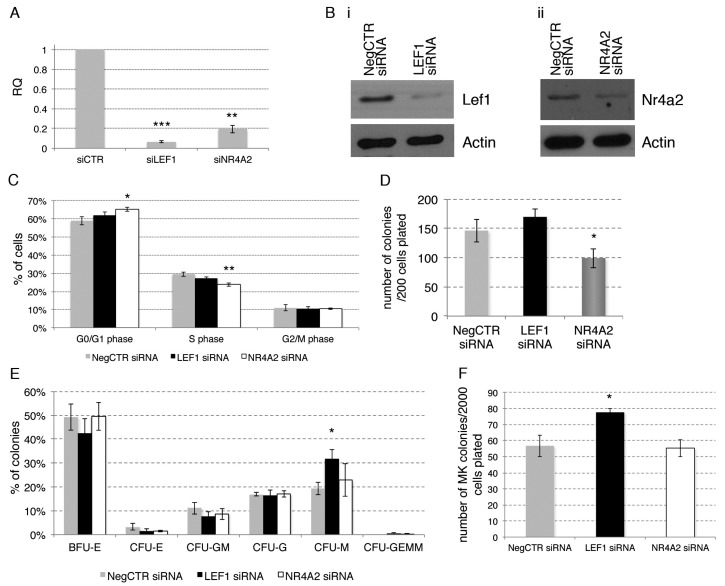

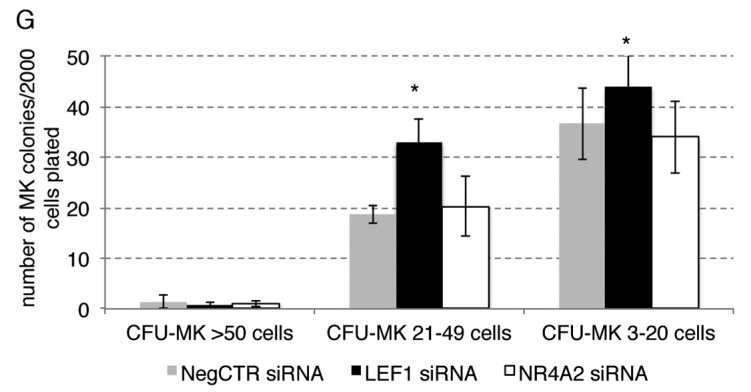
Effects of LEF1 and NR4A2 silencing on CD34+ hematopoietic progenitor cells proliferation and clonogenic activity. (**A**) Detection of *LEF1* and *NR4A2* expression levels by RT-qPCR in NegCTR siRNA, LEF1 siRNA and NR4A2 siRNA-transfected CD34+ cells at 24 h post-nucleofection; (**B**) Western Blotting analysis of Lef1 (**i**) and Nr4a2 (**ii**) protein levels in LEF1 siRNA (**i**) or NR4A2 siRNA (**ii**) compared to NegCTR siRNA-transfected CD34+ cells at 24 h after the last of two nucleofection cycles. Actin protein levels are reported as loading control; (**C**) Statistical analysis results (mean ± SEM; *n* = 3) for the percentage of cells in the different cell cycle phases performed by propidium iodide staining 24 h post-nucleofection; (**D**,**E**) Methylcellulose clonogenic assay results (mean ± SEM; *n* = 3). The colony scoring results are reported as total number of colonies grown from 200 cells plated (**D**) and percentages of each colony type (**E**); Colonies were scored according to the manufacturer’s guidelines; (**F**,**G**) Megakaryocyte clonogenic assay results (mean ± SEM; *n* = 3) in terms of megakaryocyte colony number (**F**) and size (**G**). Values are reported as number of megakaryocyte colonies for 2000 plated cells. Error bars in the graphs represent SEM. * *p* ≤ 0.05, ** *p* ≤ 0.01 and *** *p* ≤ 0.001 vs. NegCTR siRNA sample. Abbreviations: BFU-E, burst forming unit-erythroid; CFU-E, colony forming unit-erythroid; CFU-GM, colony forming unit-granulocyte/monocyte; CFU-G, colony forming unit-granulocyte; CFU-M, colony forming unit-monocyte; CFU-GEMM, colony forming unit-granulocyte/erythroid/monocyte/megakaryocyte; MK, megakaryocyte; CFU-MK, colony forming unit-megakaryocyte; *n* = number of experiments.

In a set of 3 independent experiments performed by using different HD-derived CB units, CD34+ cells were transfected with *LEF1* or *NR4A2*-targeting siRNAs (Table S2) and with a non-targeting siRNA as a negative control. The expression levels of LEF1 and NR4A2 in siRNA-treated cells (LEF1 siRNA and NR4A2 siRNA, respectively) and in non-targeting siRNA-treated cells (NegCTR siRNA) were evaluated by Real Time RT-qPCR (Figure 5A) and western Blot (Figure 5B) at 24 h post-nucleofection.

To assess the effects of LEF1 and NR4A2 silencing on CD34+ cells proliferation, a flow cytometric analysis of cell cycle distribution by PI staining was performed at 24 h post-nucleofection (Figure 5C). Cell cycle analysis demonstrated that, even though to a small extent, NR4A2 silencing restrains G1-to-S phase cell cycle progression, as shown by the increased fraction of cells in G1 phase and the decreased fraction of cells in S phase. In contast, LEF1 silencing did not affect cell cycle progression (Figure 5C).

Next, we assessed the effects of LEF1 and NR4A2 silencing on HPCs clonogenic efficiency and commitment in methylcellulose and collagen-based semisolid media (Figure 5D–G). Methylcellulose assay highlighted a remarkable reduction of the clonogenic ability in NR4A2 siRNA CD34+ cells compared with NegCTR siRNA sample (Figure 5D), while the colony scoring did not unveil any significant modulation in BFU-E, CFU-E, CFU-G, CFU-M and CFU-GM colonies (Figure 5E). In contrast, LEF1 silencing did not affect the CD34+ cells clonogenic ability (Figure 5D), while leading to a remarkable increase of the fraction of monocyte colonies (Figure 5E).

We also examined the effects of LEF1 and NR4A2 silencing on the megakaryocyte commitment by plating NegCTR siRNA, LEF1 siRNA and NR4A2 siRNA samples in a collagen-based semisolid medium (Figure 5E,F).

Collagen-based clonogenic assay unveiled that LEF1 siRNA cells give rise to a higher number of CFU-MKs in comparison with control (Figure 5F). Moreover, CFU-MKs scoring based on the size of the colonies showed that both medium (21–49 cells) and small (3–21 cells) colonies were significantly affected by LEF1 silencing. Due to the very low frequency of large (>50 cells) CFU-MKs generated, we were unable to detect any difference in their frequency between LEF1siRNA and NegCTR siRNA. (Figure 5G). Medium and large CFU-MKs are originated by primitive/immature megakaryocyte progenitors endowed of a high proliferative potential, whereas small CFU-MKs are derived from the most mature megakaryocyte progenitors. Therefore, megakaryocyte clonogenic assay data suggest that LEF1 silencing might affect both the early and the late steps of megakaryopoiesis. In contrast, we did not detect any significant effect of NR4A2 silencing on CFU-MK growth (Figure 5F,G).

These results suggest that the silencing of NR4A2 partially impairs the proliferation and the clonogenic ability of CD34+ HPCs cells, while LEF1 knockdown favours the HPCs commitment toward the monocyte/macrophage and megakaryocyte lineages.

### 2.6. Effects of LEF1 and NR4A2 on the HPCs Lineage Choice

**Figure 6:**
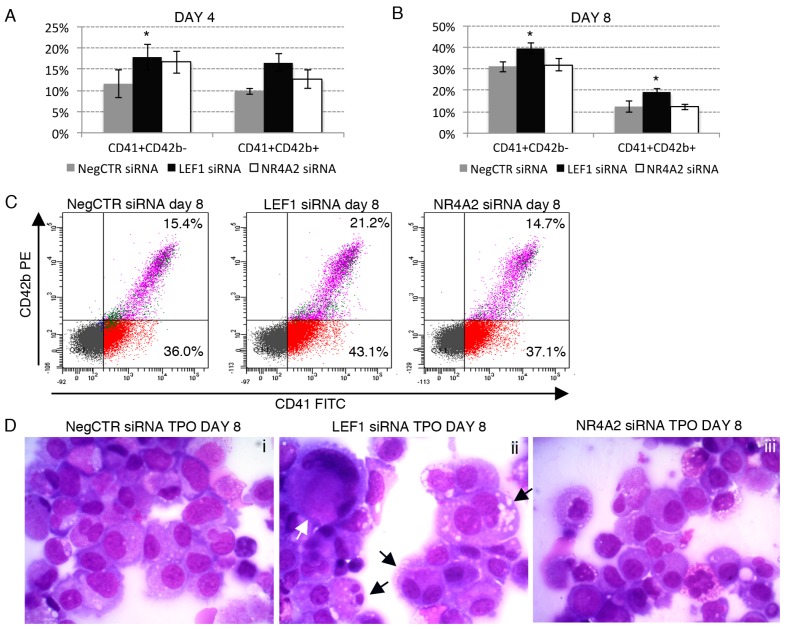

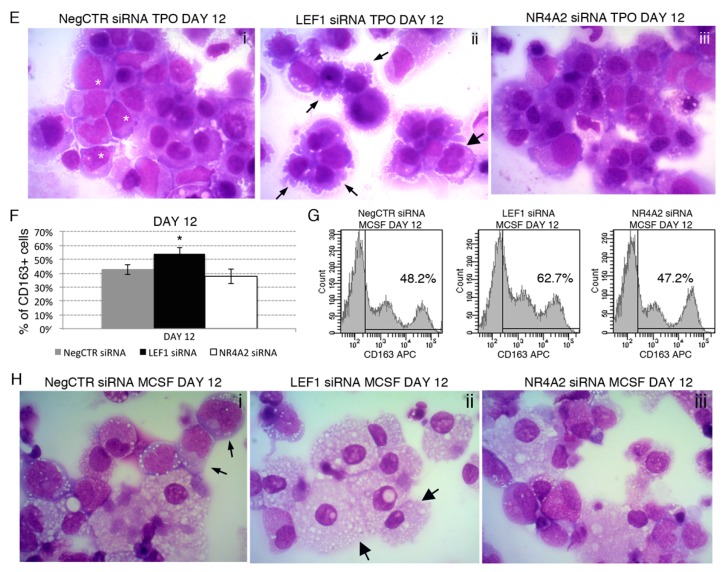
Effects of LEF1 and NR4A2 silencing on megakaryocyte and macrophage differentiation. (**A**,**B**) Flow cytometric analysis (mean ± SEM; *n* = 3) of CD41 and CD42b expression at day 4 (**A**) and day 8 (**B**) of TPO-driven megakaryocyte unilineage culture post-nucleofection; (**C**) Representative dot plots for the flow cytometric detection of CD41 and CD42b differentiation markers at day 8 of megakaryocyte unilineage culture post-nucleofection; (**D**,**E**) Morphological analysis of NegCTR siRNA (**i**), LEF1 siRNA (**ii**) and NR4A2 siRNA (**iii**) cells after May–Grunwald–Giemsa staining at day 8 (**D**) and day 12 (**E**) of megakaryocyte unilineage culture post-nucleofection in a representative experiment. White asterisks indicate megakaryoblasts, that are immature cells of the megakaryocyte lineage characterized by a higher nucleus-to-cytoplasm ratio compared to mature megakaryocytes. The white arrow highlights a large megakaryocyte. Black thick arrows indicate polyploid megakaryocytes; black thin arrows indicate cells with cytoplasmic blebs, which are a feature of the megakaryocyte maturation in vitro. Magnification, ×1000; (**F**) Flow cytometric detection (mean ± SEM; *n* = 3) of the CD163 marker at day 12 of MCSF-driven monocyte/macrophage unilineage culture post-nucleofection; (**G**) Representative histograms for the flow cytometric detection of CD163 marker at day 12 of monocyte/macrophage unilineage culture post-nucleofection; (**H**) Morphological analysis of NegCTR siRNA (**i**), LEF1 siRNA (**ii**) and NR4A2 siRNA-transfected cells (**iii**) after May–Grunwald–Giemsa staining at day 12 of monocyte/macrophage unilineage culture post-nucleofection in a representative experiment. Thick arrows indicate macrophages; thin arrows indicate monocytes. Magnification, ×1000. Data are from *n* = 3 independent experiments performed with different healthy donor-derived cord blood units. * *p* ≤ 0.05 compared to NegCTR siRNA sample. Abbreviations: TPO, thrombopoietin; MCSF, monocyte colony-stimulating factor.

To further clarify the role of LEF1 and NR4A2 in HPCs lineage choice, we assessed the effects of LEF1 and NR4A2 silencing in HPCs lineage commitment and differentiation in a similar way as reported for miR-34a-5p overexpression experiments (Figure 6 and Figure S3).

Our data revealed that LEF1 silencing promotes the megakaryocyte differentiation. Indeed, flow cytometry data showed an increased fraction of CD41+CD42b− (immature megakaryocytes) and CD41+CD42b cells (mature megakaryocytes) in LEF1 siRNA sample in comparison to NegCTR siRNA at 4 and 8 days of TPO-induced megakaryocyte differentiation (Figure 6A–C). In keeping with flow cytometry data, morphological analysis of MGG-stained cytospins at 8 and 12 days of culture (Figure 6D,E) unveiled that LEF1 siRNA samples were enriched in more mature megakaryocytes, such as large and polyploid cells and megakaryocytes displaying cytoplasmic blebs that are features of the megakaryocyte differentiation in vitro (Figure 6Dii,Eii) in comparison with NegCTR siRNA sample (Figure 6Di,Ei). In contrast, no significant differences in megakaryocyte maturation was detected in NR4A2 siRNA (Figure 6Diii,Eiii) samples compared with controls (Figure 6Di,Ei) cultured under the same conditions.

Additionally, a remarkable increase in the macrophage differentiation emerged in LEF1 siRNA sample compared with NegCTR siRNA and NR4A2 siRNA samples under monocyte/macrophage-specific culture conditions (Figure 6F–H), as assessed by the increased CD163-positive macrophage population detected by flow cytometry (Figure 6F,G) and the augmented macrophage-to-monocyte ratio seen by morphological analysis (Figure 6H).

Furthermore, the effects of LEF1 and NR4A2 silencing on erythroid and granulocyte differentiation of CD34+ cells were assayed (Figure S3). NegCTR siRNA, LEF1 siRNA and NR4A2 siRNA samples displayed a similar composition in terms of early CD36+GPA−, intermediate CD36+GPA+ and late CD36−GPA+ erythroid cells, as shown by flow cytometry at 8 and 12 days of erythroid differentiation (Figure S3A,B). In line, both NegCTR siRNA, LEF1 siRNA and NR4A2 siRNA samples were as similar mixture of erythroblasts and reticulocytes that have already extruded the nucleus at 12 days of culture with EPO (Figure S2C). Finally, we did not detect any difference between LEF1/NR4A2 siRNA and NegCTR siRNA samples during the GCSF-driven granulocyte differentiation, as assessed by flow cytometric analysis of myeloperoxidase at 8 and 12 days (Figure S3D) and morphological analysis of MGG-stained cytospins at 12 days, the latter displaying a mixed population of promyelocytes, band neutrophils and mature neutrophils in all the samples (Figure S3E).

## 3. Discussion

We recently performed a comparative analysis of the gene and miRNA expression profiles of CD34+ HPCs from PMF patients and HDs [12]. Since miRNA profiling data highlighted the overexpression of miR-34a-5p in PMF cells compared with their normal counterparts [12], in the present study we wondered whether and how miR-34a-5p could affect HPCs fate decision and could have a role in PMF pathogenesis.

We firstly add novel insights into the PMF-related upregulation of miR-34a-5p by showing that the increased expression of miR-34a-5p in CD34+ cells from PMF patients is a feature shared by *JAK2*-mutated, *MPL*-mutated and *CALR*-mutated PMF patients, while it seems to be negligible in triple-negative PMF patients. However, since *JAK2*, *MPL* and *CALR* mutations all converge toward the constitutive activation of JAK/STAT pathway, our data suggest that the deranged JAK/STAT signaling could underlie the upregulation of miR-34a-5p in PMF CD34+ cells.

Interestingly, the activation of MEK1/2-ERK1/2 signaling pathway triggers the expression of miR-34a-5p in K562 cells during the phorbol 12-myristate 13-acetate (PMA)-induced megakaryocytic differentiation [19]. Interestingly, increased ERK1/ERK2 phosporylation levels have been detected in CD34+ cells from PMF patients [20]. Therefore, the deranged activation of the MEK/ERK cascade in CD34+ cells from PMF patients [20] together with JAK/STAT signaling, could contribute to the upregulation of miR-34a-5p.

Here we demonstrate that the overexpression of miR-34a-5p in normal CD34+ HPCs slightly but significantly constrains cell cycle progression at G1-to-S phase transition and impairs the clonogenic ability. Furthermore, we showed that the enforced expression of miR-34a-5p in CD34+ HPCs favours their differentiation toward the megakaryocyte and monocyte/macrophage lineages. In contrast, the erythroid and granulocyte lineages were not significantly affected by miR-34a-5p overexpression. Our data are in line with those by Navarro et al., showing that in K562 cells the overexpression of miR-34a-5p arrests cell cycle progression at G1-to-S phase transition and enhances the PMA-induced megakaryocytic differentiation [13]. Remarkably, all the effects of the miR-34a-5p forced expression we detected in the HPCs (namely, the inhibition of G1-to-S phase cell cycle progression and the increased megakaryocyte and macrophage differentiation) are reminiscent of those induced by the downregulation of MYB in these cells [17,18]. Indeed, MYB has been already reported as a miR-34a-5p target [13] whose downregulation at least partially accounts for the miR-34a-5p-driven enhanced megakaryopoiesis.

In order to further unravel the molecular mechanisms underlying the miR-34a-5p-mediated perturbation of the HPCs proliferation and differentiation and to gain insights into its role in PMF pathogenesis, we profiled by microarrays the gene expression changes induced by miR-34a-5p overexpression in CD34+ cells. Even though neither microarray nor RT-qPCR detected a downregulation of the MYB transcript, Myb protein levels strongly decreased upon miR-34a-5p overexpression in HPCs. Therefore, we hypothesised that the miR-34a-5p-mediated downregulation of MYB could be ascribed to a miR-34a-5p-mediated inhibition of MYB mRNA translation rather than mRNA degradation. Remarkably, these data, together with those from MYB silencing experiments we previously published [17,18] further strenghten the idea that MYB is a key player in miR-34a-5p-driven perturbation of HPCs proliferation and differentiation.

We next focused our attention on LEF1 and NR4A2 as transcriptional regulators downregulated in HPCs after miR-34a-5p overexpression as well as in HPCs from PMF patients and therefore potentially involved in PMF pathogenesis. Worth to be mentioned, both LEF1 and NR4A2 were experimentally validated as miR-34a-5p targets [15,16]. We firstly analysed their expression kinetics during CD34+ cells differentiation. Interestingly, miR-34a-5p and *LEF1* display an anticorrelated expression trend during both the megakaryocyte and the monocyte/macrophage differentiation, further suggesting that LEF1 targeting could be an important mechanism for miR-34a-5p-mediated effects in CD34+ cells differentiation.

We subsequently moved to the role of LEF1 and NR4A2 in HPCs proliferation and lineage choice by their knockdown in normal CD34+ cells. We showed that the downregulation of NR4A2 can at least partially account for the antiproliferative effect of miR-34a-5p, together with the miR-34a-5p-driven knockdown of MYB [17].

NR4A2 (NURR1) belongs to the family of NR4A orphan nuclear hormone receptors together with NR4A1 and NR4A3 (NURR77 and NOR1, respectively). NR4A family members act as transcription factors and bind the consensus sequences AAGGTCA as monomers or TGATATTTX6AAAGTCCA as homo/heterodimers to regulate the expression of their target genes [21,22,23]. In contrast with its role in maintaining the HSCs quiescence while restraining them to enter cell cycle [24,25], here we demonstrate that NR4A2 favours the HPCs proliferation, while its silencing partially constrains G1-to-S phase cell cycle progression. This is in agreement with the growth-inhibitory effect of NR4A2 silencing detected in breast cancer [26], colon cancer [16] and intestinal epithelial cells [27] and suggests a different role for NR4A2 in HSCs and HPCs proliferation. However, it is likely that the constitutive activation of the JAK/STAT pathway caused by the mutations in *JAK2*, *MPL* and *CALR* genes in PMF patients [8,28,29] could overcome the growth-inhibitory effects of miR-34a-5p forced expression and NR4A2 knockdown we highlighted in normal HPCs.

With the aim to unravel the role of LEF1 in HPCs proliferation and lineage choice, we also silenced LEF1 expression in CD34+ cells. We demonstrated that, while not affecting CD34+ cells proliferation, LEF1 downregulation phenocopies the effects of miR-34a-5p overexpression on the HPCs fate decision. Indeed, methylcellulose and collagen-based clonogenic assays, together with the immunophenotypic and morphological analysis of differentiation, highlighted a remarkable increase of the megakaryocyte and the macrophage differentiation in LEF1-silenced compared to NegCTR siRNA-transfected cells.

LEF1 is a transcription factor member of the T-cell factor/lymphoid enhancer factor (TCF/LEF) family which acts in association with β-catenin in Wnt canonical signaling pathway [30]. Macaulay et al showed that Wnt signaling affects the proliferation and maturation of mouse fetal liver-derived megakaryoytes [31]. In addition, the group of Ballestar recently demonstrated that the miR-34a-5p-mediated downregulation of LEF1 is essential for the C/EBPα-mediated transdifferentiation of pre-B lymphoid cells into functional macrophages [32]. This is consistent with our data that uncover the function of miR-34a-5p/*LEF1* axis in enforcing the macrophage differentiation of HPCs.

One of the main features of PMF patients is the abnormal number of dysplastic megakaryocytes that accumulate in the BM and release proinflammatory and profibrotic cytokinesby fostering the development of BM fibrosis. Together with the megakaryocytes, also monocytes play a key role in the overwhelming release of proinflammatory and profibrotic mediators (e.g., TGF-β, PDGF, bFGF and VEGF) that act on stromal cells and prompt the development of PMF-associated abnormalities of the BM architecture, such as BM fibrosis and osteosclerosis [6,7,33,34]. Indeed, the elucidation of the molecular mechanisms underlying the deranged megakaryopoiesis and the *inflammation storm* [4] in PMF patients is of paramount importance to identify novel therapeutic targets to constrain it and, therefore, to reverse fibrosis [33]. Notably, even though it does not affect the HPCs differentiation toward the monocyte/macrophage lineage, NR4A2 expression is reported to be strongly induced in macrophages in response to proinflammatory stimuli (e.g., LPS and TNF-α) and acts by reducing the synthesis of several cytokines and chemokines to constrain the macrophage proinflammatory phenotype. By contrast, the knockdown of NR4A2 prompts the synthesis of proinflammatory mediators such as IL1B, IL8 and MCP1 in macrophages [35]. Therefore it would be of interest to investigate whether the downregulation of NR4A2 could account for the proinflammatory macrophage phenotype in PMF patients and ultimately contribute to the development of BM fibrosis and osteosclerosis [6,7,33,34]. Indeed, the downregulation of NR4A2 was recently detected in fibrotic liver compared with the normal counterpart and was recently associated with the hepatic stellate cells activation and the liver fibrosis onset [36].

**Figure 7:**
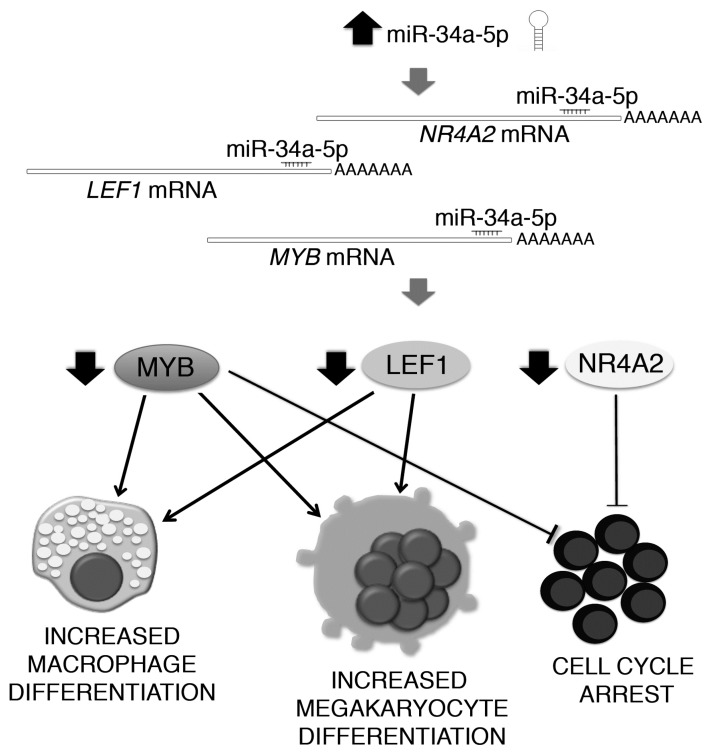
Schematic illustration of the mechanism through which miR-34a-5p affects the hematopoietic progenitor cells proliferation and commitment. miR-34a-5p targets *MYB*, *LEF1* and *NR4A2* transcripts in HPCs, therefore leading to the downregulation of MYB, LEF1 and NR4A2 proteins (ellipses). By targeting MYB, miR-34a-5p impairs the hematopoietic progenitor cells proliferation and enhances the megakaryocyte and macrophage differentiation. In addition, miR-34a-5p targets LEF1 mRNA for degradation. LEF1 downregulation further enhances the hematopoietic progenitor cells commitment towards the megakaryocyte and macrophage lineages, while the miR-34a-5p-driven downregulation of NR4A2 negatively interferes with cell cycle progression. Therefore, miR-34a-5p restrains hematopoietic progenitor cells proliferation and favours their commitment toward the megakaryocyte and macrophage lineages by targeting LEF1 and NR4A2, together with MYB.

As a whole, our data shed some light on the role of miR-34a-5p in HPCs proliferation and fate decision (Figure 7). Indeed, miR-34a-5p is able to negatively interfere with HPCs proliferation by targeting NR4A2, together with MYB [13,17]. Additionally, miR-34a-5p enhances the HPCs commitment toward the megakaryocyte and macrophage lineages by targeting LEF1 and MYB [13,17,18].

Interestingly, our results suggest that the increased expression of miR-34a-5p in PMF HPCs could contribute to the abnormal megakaryopoiesis seen in PMF patients through the downregulation of LEF1 and MYB [13,17]. In contrast, the antiproliferative effect of the miR-34a-5p/NR4A2 axis is likely overcome by the constitutive activation of the JAK/STAT signaling pathway that drives the myeloproliferation in PMF patients [37].

Altogether, our data demonstrated that miR-34a-5p is an important regulator of HPCs proliferation and fate decision due to its ability to block the HPCs proliferation through the downregulation of NR4A2 and MYB and to promote the megakaryocyte and macrophage differentiation by targeting LEF1 and MYB (Figure 7). In addition, our study provides some intriguing insights into how the aberrant expression of miR-34a-5p could underlie the skewed megakaryopoiesis and other traits of PMF pathogenesis.

## 4. Materials and Methods

### 4.1. Ethics Statement

Human CD34+ cells were purified upon donor’s informed written consent from healthy donor-derived umbilical Cord Blood (CB) samples, collected after normal deliveries, according to the institutional guidelines for discarded material (Clearance of Ethical Commitee for Human experimentation of Modena: Secretary office Saverio Santachiara, santachiara.saverio@policlinico.mo.it, approval date: 18 January 2005; approval file number # 793/CE).

### 4.2. miR-34a-5p Expression Analysis in PMF and HD CD34+ Cells

The raw Affymetrix data (CEL files) previously published [12] for CD34+ cells isolated from PMF patients (*n* = 38) and HDs (*n* = 30; *n* = 14 from BM and *n* = 16 from peripheral blood, PB) were downloaded from the NCBI’s Gene Expression Omnibus (GEO) public repository [38] (http://www.ncbi.nlm.nih.gov/geo; series GSE53482). The probe level data were normalized and converted into expression values by using the robust multiarray average (RMA) procedure in Partek^®^ Genomics Suite^®^ software, version 6.6 Copyright^©^, 2016 (Partek Inc., St. Louis, MO, USA; http://www.partek.com). The characterization of PMF samples was implemented by adding the driver mutation carried by each PMF patient (i.e., mutation in *JAK2*, *MPL* or *CALR*). PMF samples were therefore classified into *JAK2*-mutated (*n* = 20), *MPL*-mutated (*n* = 3), *CALR*-mutated (*n* = 9) and triple-negative (*n* = 6). RMA-normalized miR-34a-5p expression data were analysed through a *t*-test statistic of the pair-wise comparisons.

### 4.3. Human CD34+ Hematopoietic Progenitor Cells (HPCs) Purification

Umbilical CB samples were collected after normal deliveries, according to the institutional guidelines for discarded material. CB CD34+ cells were purified as previously described [39]. After immunomagnetic separation, CD34+ cells were seeded in 24-well plates at 5 × 10^5^/mL in serum-free medium SYN-H (ABCell-Bio, Paris, France) supplemented with stem cell factor (SCF) (50 ng/mL), Flt3-ligand (Flt3L) (50 ng/mL), TPO (20 ng/mL), IL-6 (10 ng/mL) and IL-3 (10 ng/mL) (all from Miltenyi Biotec, Bergisch Gladbach, Germany).

### 4.4. miR-34a-5p, LEF1 and NR4A2 Kinetics during the Erythroid, Megakaryocyte, Granulocyte and Mono-Macrophage Differentiation of CD34+ HPCs

After immunomagnetic separation, CD34+ cells were seeded in 24-well plates at a density of 5 × 10^5^/mL in IMDM added with 20% BIT serum substitute (bovine serum albumin, insulin, and transferrin; StemCell Technologies, Vancouver, BC, Canada) in order to set up erythroid (SCF 10 ng/mL and EPO 0.4 U/mL, adapted from Tenedini et al. [39]), megakaryocyte (TPO 50 ng/mL, SCF 10 ng/mL, adapted from Tenedini et al. [39]), granulocyte (GCSF 25 ng/mL, SCF 10 ng/mL, adapted from Kandilci et al. [40]) and monocyte/macrophage [40] (MCSF 100 ng/mL, SCF 20 ng/mL, IL6 20 ng/mL and FLT3L 50 ng/mL, all cytokines from Miltenyi Biotec, Bergisch Gladbach, Germany) unilineage cultures. The medium was replaced every 3 days. CD34+ cells differentiation was monitored by morphological analysis of MGG-stained cytospins and by flow-cytometric analysis of differentiation marker expression. miR-34a-5p, LEF1 and NR4A2 expression levels were detected by Real-Time RT-qPCR at different time points (i.e., day 0, 4, and 8) after the seeding of cells in erythroid, megakaryocyte, granulocyte or monocyte/macrophage unilineage cultures.

### 4.5. Nucleofection of CD34+ Cells

Human CD34+ cells were transfected by using the 4D-Nucleofector^TM^ System (Lonza, Allendale, NJ, USA) as previously reported [12]. Briefly, starting from the day after CD34+ cells purification, each sample was electroporated twice, once every 24 h, with 3 µg of mirVana^TM^ miR-34a-5p mimic or mirVana^TM^ miRNA mimic Negative Control (NegCTR mimic) (Thermo Fisher Scientific, Waltham, MA, USA). For each electroporation, 4 × 10^5^ CD34+ cells were resuspended in 100 µL of P3 Primary Cell Solution (Lonza) containing 3 µg of miR-34a-5p mimic or NegCTR mimic and pulsed with the program DS112. 

Similarly, small interfering RNAs (siRNAs) transfection was performed as previously reported [12]. In order to significantly reduce the risk of siRNA-mediated off-target effects, we selected Ambion^®^ Silencer^®^ Select siRNAs (Thermo Fisher Scientific, Waltham, MA, USA), which are next-generation siRNAs designed to minimize by up to 90% the off-target activity therefore providing the highest specificity and consistent phenotypic data. Indeed, we performed preliminary experiments for LEF1 silencing to assess whether three different siRNAs (Ambion^®^ Silencer^®^ Select Pre-designed siRNAs #s27616, #s27617 and #s27618) resulted in similar knockdown efficiency and phenotipic effects. Overlapping results in terms of LEF1 knockdown efficiency and LEF1 silencing effects were collected. Therefore, the LEF1 silencing experiments reported here were performed by using the Ambion^®^ Silencer^®^ Select Pre-designed siRNA #s27616 (Table S2).

Briefly, CD34+ cells were nucleofected three times, once every 24 h, with siRNAs targeting human *LEF1 or NR4A2* mRNAs (Table S2) (Thermo Fisher Scientific, Waltham, MA, USA) by using the electroporation protocol DS112 mentioned above for mimics transfection. To exclude non-specific effects caused by the siRNA nucleofection, one sample transfected with a non-targeting siRNA (NegCTR siRNA; Thermo Fisher Scientific, Waltham, MA, USA) for each experiment was included.

Nucleofected CD34+ cells were maintained in serum-free culture conditions (SYN-H, ABCell-Bio, Paris, France) in the presence of Flt3-ligand (Flt3L) (50 ng/mL), SCF (50 ng/mL), TPO (20 ng/mL), IL-3 (10 ng/mL) and IL-6 (10 ng/mL) (Miltenyi Biotec, Bergisch Gladbach, Germany).

For liquid culture differentiation assays, 24 h after the last nucleofection (hereafter reported as post-nucleofection) CD34+ cells were plated (5 × 10^5^/mL) in IMDM added with 20% BIT serum substitute (bovine serum albumin, insulin, and transferrin; StemCell Technologies, Vancouver, BC, Canada), in order to set up erythroid (SCF 10 ng/mL and EPO 0.4 U/mL, adapted from Tenedini et al. [39]), megakaryocyte (TPO 50 ng/mL, SCF 10 ng/mL, adapted from Tenedini et al. [39]), granulocyte (GCSF 25 ng/mL, SCF 10 ng/mL, adapted from Kandilci et al. [40]) and monocyte/macrophage [40] (MCSF 100 ng/mL, SCF 20 ng/mL, IL6 20 ng/mL and FLT3L 50 ng/mL, all cytokines from Miltenyi Biotec, Bergisch Gladbach, Germany).

### 4.6. Methylcellulose- and Collagen-Based Clonogenic Assays

Methylcellulose-based assay was carried out by plating 2000 CD34+ cells in StemMACS HSC-CFU medium (cat# 130-091-280; Miltenyi Biotec, Bergisch Gladbach, Germany). Megakaryocyte colony forming units (CFU-MK) were assayed in collagen-based medium, by using a commercial MK assay detection kit (MegaCult-C, StemCell Technologies, Vancouver, BC, Canada) as previously described [40]. CFU-MK colonies were screened for their size according to the following categories: (a) small colonies, that are made of 3–21 cells and originate fromlate megakaryocyte progenitors with a low proliferative capacity; (b) medium colonies, which are made of 21–49 cellsand are generated by intermediate megakaryocyte progenitors and (c) large colonies, which are made of more than 50 cells and springfrom early, immature MK progenitors endowed of a high proliferative potential.

### 4.7. Cell Cycle Analysis

Cell cycle analysis was performed at 24 h after the last nucleofection by flow cytometry according to Nicoletti et al. [41].

### 4.8. Morphological and Immunophenotypic Analysis

Differentiation of CD34+ cells was assessed by morphological analysis of May-Grunwald-Giemsa-(MGG)-stained cytospins at 4, 8 and 12 days post-nucleofection and by flow-cytometric analysis of differentiation markers expression (CD34, CD38, CD71, CD36, Glycophorin A (GPA), myeloperoxydase (MPO), CD163, CD41 and CD42b) at 4, 8 and 12 days post-nucleofection.

Micrographs of the MGG-stained cytospins were taken by means of anAxioscopeA1 microscope provided with AxioCam ERc 5S Digital Camera (Carl Zeiss MicroImaging Inc.; Thornwood, NY, USA). The following monoclonal antibodies (MoAbs) were used for flow cytometric analysis: fluorescein isothiocyanate (FITC)-conjugated mouse anti-CD34 MoAb, phycoerythrin (PE)-conjugated mouse anti-human CD38 MoAb, FITC-conjugated mouse anti-human CD36 MoAb, allophycocyanin (APC)-conjugated mouse anti-human CD71 MoAb, APC-conjugated mouse anti-human CD163 MoAb (all from Miltenyi Biotec, Bergisch Gladbach, Germany), FITC-conjugated mouse anti-CD41 MoAb, PE-conjugated mouse anti-human CD42b MoAb, PE-conjugated mouse anti-human GPA MoAb (all from Dako; Milano, Italia; http://www.dako.com) and FITC-conjugated mouse anti-human MPO MoAb (from BD Biosciences; San Jose, CA, USA). Flow cytometric analyses were performed by means of a BD FACSCanto II (BD Biosciences; San Jose, CA, USA). At least 10,000 events for sample were acquired.

### 4.9. RNA Extraction

Total cellular RNA, including small RNAs, was isolated from 2 × 10^5^ cells for each sample using the Qiagen miRNeasy^**®**^ Micro RNA isolation kit (Qiagen, Hilden, Germany) following the manufacturer’s recommendations. For each sample RNA concentration and integrity were assessed by NanoDrop ND-1000 spectrophotometer (NanoDrop Technologies; Wilmington, DE, USA) and Agilent 2100 Bioanalyzer (Agilent Technologies; Waldbrunn, Germany), respectively.

### 4.10. Gene Expression Profiling (GEP)

GEP was performed on RNA samples isolated from miR-34a-5p mimic and NegCTR mimic CD34+ cells at 24 h after two nucleofections from 3 independent experiments.

GEP was performed by means of HG-U219 Array Strips (Affymetrix; Santa Clara, CA, USA) as previously described [18].

Robust multiarray average (RMA)-normalized data were analysed through the Analysis of Variance (ANOVA) module provided by the Partek^®^ Genomics Suite^®^ software, version 6.6 Copyright^©^, 2016 (Partek Inc., St. Louis, MO, USA; http://www.partek.com). A fold change (FC) contrast ≥2 (*p* < 0.05) was set as cutoff in order to identify the differentially expressed genes (DEGs) in miR-34a-5p mimic vs. NegCTR mimic samples. All the GEP data have been deposited in the GEO public repository [38] (http://www.ncbi.nlm.nih.gov/geo; series GSE89156).

### 4.11. Quantitative Reverse Transcription Polymerase Chain Reaction (RT-qPCR)

Total RNA reverse transcription and TaqMan Real Time RT-qPCR were performed as previously described [18] Relative expression levels were achieved by using the comparative cycle threshold (*C*_t_) method of relative quantitation using GAPDH as the housekeeping gene. To normalize data, ΔΔ*C*_t_ was calculated for each sample using the mean of its Δ*C*_t_ values subtracted from the mean Δ*C*_t_ value measured in the NegCTR mimic/NegCTR siRNA sample, set as a calibrator; relative quantitation (RQ) value was expressed as 2^−ΔΔ*C*t^. LEF1/NR4A2 relative expression kinetics during erythroid, megakaryocyte, granulocyte or monocyte/macrophage differentiation were similarly calculated by setting CD34+ cells as calibrator.

The miR-34a-5p expression levels were detected by RT-qPCR by means of TaqMan MicroRNA assays specific for miR-34a-5p and U6 snRNA, respectively (Thermo Fisher Scientific, Waltham, MA, USA). Five ng of total RNA were reverse-transcribed by using miRNA-specific looped-primers. Real Time RT-qPCR was performed by means of a 7900HT Fast Instrument (Applied Biosystems, Foster City, CA, USA). U6 snRNA was set as housekeeping control to assess miR-34a-5p Relative Quantity (RQ).

### 4.12. Western Blot

c-Myb, Lef1 and Nr4a2 protein levels in miR-34a-5p-overexpressing and/or NR4A2/LEF1-silenced CD34+ cells were detected by Western blot analysis as already described [17], with some modifications. Briefly: cells were harvested, washed twice with ice-cold PBS and lysed (3 × 10^5^ cells/20 µL of lysis buffer) in 50 mM Tris (tris(hydroxymethyl)aminomethane)-Cl (pH 7.4), 150 mM NaCl, 1% Nonidet P-40, 10 mM KCl, 1 mM EDTA, 20 mM NaF, 0.25% Na doexycholate, 5 mM dithiothreitol (DTT) and protease inhibitors (Complete, catalog #1697498, Roche, Indianapolis, IN, USA). Total cellular lysates (20 μg for each sample) were loaded onto 7.5% SDS-polyacrylamide gel and blotted as described. To control loading and transfer, after transfer the membranes were stained by Red Ponceau.

Membranes were preblocked in blocking solution, 5% milk in 0.1% TBST for 1 h at room temperature (RT), and therefore incubated overnight at 4 °C with one of the following antibodies: (a) 1:1000 dilution of mouse monoclonal anti-Myb primary antibody (clone 1-1, catalog #27282, Upstate, Lake Placid, NY, USA); (b) 1:1000 dilution of rabbit polyclonal anti-Lef1 primary antibody (catalog #ab124271, Abcam, Cambridge, UK); (c) 1:500 dilution of mouse monoclonal anti-Nr4a2 primary antibody (catalog #ab55769, Abcam, Cambridge, UK), or incubated at RT for 1 h with 1:2000 dilution of rabbit polyclonal anti–actin primary polyclonal antibody (catalog #PA1-16889, Thermo Fisher Scientific). After 3 washes with TBST, blots were incubated with HRP-conjugated goat anti-mouse (1:5000 dilution, catalog # sc-2005, Santa Cruz Biotechnology, Santa Cruz, CA, USA) or HRP-conjugated goat anti-rabbit (1:10,000 dilution, Pierce) secondary antibody for 1 h at RT and revealed by BM Chemiluminescence Blotting Substrate (POD) (Roche, Indianapolis, IN, USA).

### 4.13. Statistical Analysis

For both miRNA overexpression and gene silencing experiments data were collected from 3 independent biological replicates starting from different healthy donor-derived CB units. Statistical analysis was performed by using a two-tailed Student’s *t*-test for averages comparison in paired samples. Data were analyzed by using Microsoft Excel (Microsoft Office, 2008 release) and are reported as mean ± standard error of the mean (SEM). *p* < 0.05 was considered significant.

## 5. Conclusions

Our findings demonstrate that miR-34a-5p affects the HPCs proliferation and lineage choice by targeting LEF1 and NR4A2, together with MYB. Indeed, we showed that miR-34a-5p negatively interferes with HPCs proliferation through the downregulation of NR4A2 expression and enhances the HPCs commitment toward the megakaryocyte and macrophage lineages by targeting LEF1. Our study provides insights into how the upregulation of miR-34a-5p could be involved in the hyperplastic megakaryopoiesis and in some alterations of the bone marrow architecture that are a hallmark of PMF pathogenesis.

## Figures and Tables

**Table 1:** Transcripts downregulated upon hsa-miR-34a-5p overexpression in CD34+ cells.

Probeset ID	Gene Symbol	Gene Title	RefSeq Transcript ID	FC miR-34a-5p Mimic vs. NegCTR Mimic CD34+ Cells	TargetScan-Predicted miR-34a-5p Target	Modulation in PMF vs. HD CD34+ Cells
11734171_at	*PRTN3*	proteinase 3	NM_002777	−3.81	−	−
11728026_x_at	*IGLL1*	immunoglobulin lambda-like polypeptide 1	NM_020070; NM_152855	−3.22	−	−
11756600_a_at	*TPD52*	tumor protein D52	NM_001025252;NM_001025253; NM_005079	−2.93	+	−
11729643_s_at	*TPD52*	tumor protein D52	NM_001025252; NM_001025253; NM_005079	−2.89	+	−
11754659_x_at	*TPD52*	tumor protein D52	NM_001025252;NM_001025253;NM_005079	−2.69	+	−
11727965_at	*ELANE*	elastase, neutrophil expressed	NM_001972	−2.65	−	−
11715306_s_at	*AREG; AREGB*	amphiregulin /// amphiregulin B	NM_001657; XM_001125684	−2.61	+	D
11745205_s_at	*TPD52*	tumor protein D52	NM_001025252;NM_001025253; NM_005079	−2.59	+	−
11723339_at	*CTSG*	cathepsin G	NM_001911	−2.29	−	−
11726333_s_at	*LEF1*	lymphoid enhancer-binding factor 1	NM_001130713;NM_001130714;NM_001166119; NM_016269	−2.29	+	D
11718477_a_at	*STAR*	steroidogenic acute regulatory protein	NM_000349; NM_001007243	−2.24	−	−
11720051_at	*SPOCK1*	sparc/osteonectin, cwcv and kazal-like domains proteoglycan (testican) 1	NM_004598	−2.17	−	−
11718479_x_at	*STAR*	steroidogenic acute regulatory protein	NM_000349; NM_001007243	−2.17	−	−
11715245_s_at	*IGLL1*	immunoglobulin lambda-like polypeptide 1	NM_020070; NM_152855	−2.15	−	−
11729641_a_at	*TPD52*	tumor protein D52	NM_001025252; NM_001025253; NM_005079	−2.06	+	−
11725632_at	*NR4A2*	nuclear receptor subfamily 4, group A, member 2	NM_006186; NM_173171; NM_173172; NM_173173	−2.03	+	D

Abbreviations: FC, Fold Change; PMF, primary Myelofibrosis; HD, healthy donor; D, dowregulated; +, yes; −, no.

## Supplementary Materials

Supplementary materials can be found at www.mdpi.com/1422-0067/18/1/145/s1.
