# Supplementary material for: Role of miR-34a-5p in Hematopoietic Progenitor Cells Proliferation and Fate Decision: Novel Insights into the Pathogenesis of Primary Myelofibrosis
Source: Int J Mol Sci. 2017 Jan 13;18(1):145. doi: 10.3390/ijms18010145 (PMC5297778; doi:10.3390/ijms18010145)
Supplement: Supplementary file 1 [file ijms-18-00145-s001.pdf]

# Supplementary Materials: Role of miR-34a-5p in Hematopoietic Progenitor Cells Proliferation and Fate Decision: Novel Insights into the Pathogenesis of Primary Myelofibrosis

**Elisa Bianchi, Samantha Ruberti, Sebastiano Rontauroli, Paola Guglielmelli, Simona Salati, Chiara Rossi, Roberta Zini, Enrico Tagliafico, Alessandro Maria Vannucchi, Rossella Manfredini and on behalf of the AGIMM (AIRC Gruppo Italiano Malattie Mieloproliferative) Investigators**

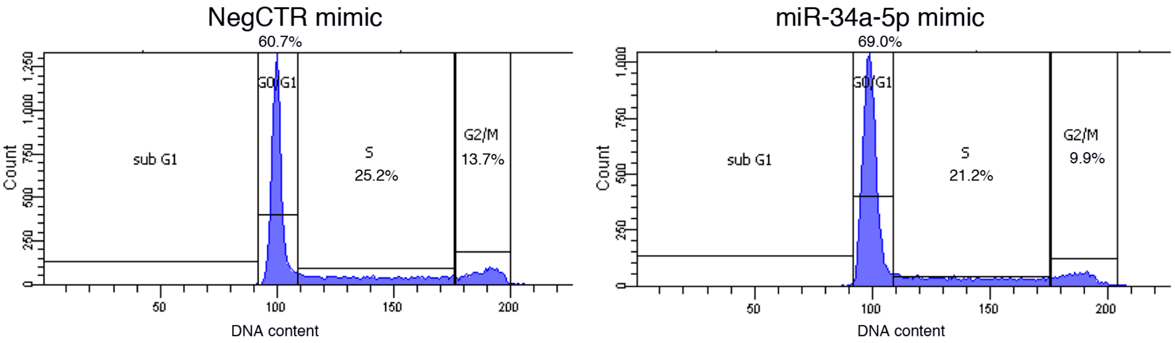


(**A**)

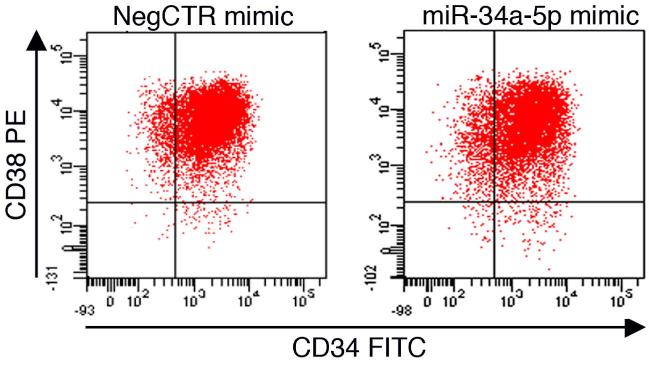


(**B**)

**Figure S1.** Effects of miR-34a-5p overexpression on CD34+ hematopoietic progenitor cells proliferation and commitment. (**A**) Representative histograms for the flow cytometric analysis of cell cycle distribution by propidium iodide staining in NegCTR mimic and miR-34a-5p mimic CD34+ cells; (**B**) Representative dot plots for the flow cytometric detection of CD34 and CD38 markers at 24 h post-nucleofection. No significant differences in the earliest CD34+CD38− stem cell population, the intermediate CD34+CD38+ hematopoietic progenitor cells fraction and the most committed CD34−CD38+ population were detected between NegCTR mimic and miR-34a-5p samples.

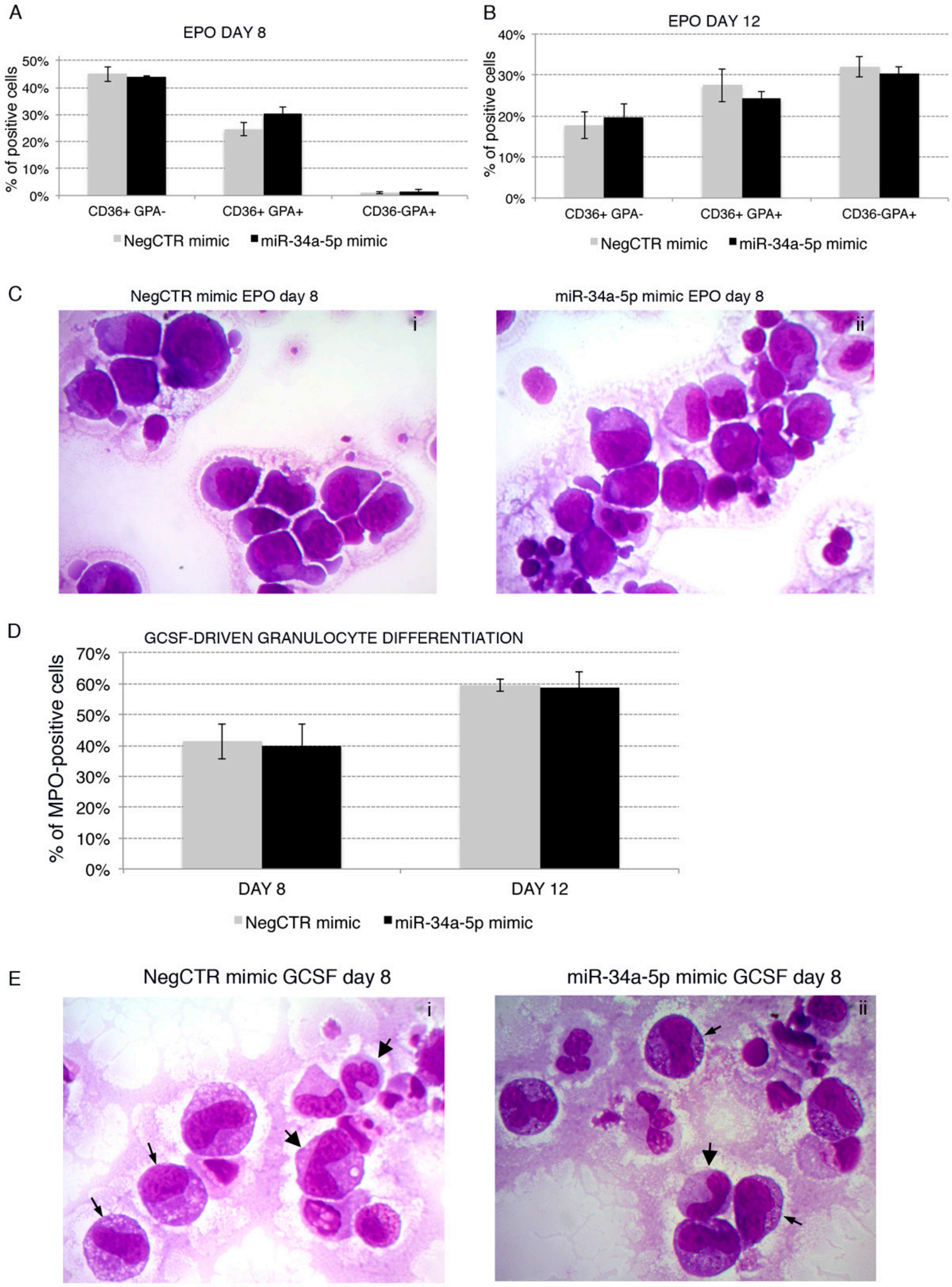


**Figure S2.** Effects of miR-34a-5p overexpression on erythroid and granulocyte differentiation. (**A**,**B**) Flow cytometric analysis (mean ± SEM; *n* = 3) of CD36 and glycophorin A (GPA) expression at day 8 (**A**) and day 12 (**B**) of EPO-driven erythroid unilineage culture post-nucleofection; (**C**) Morphological analysis of NegCTR mimic (**i**) and miR-34a-5p mimic-transfected cells (**ii**) after May–Grünwald–Giemsa staining at day 8 of EPO-induced erythroid differentiation post-nucleofection in a representative experiment. Magnification, ×1000; (**D**) Flow cytometric detection (mean ± SEM; *n* = 3) of the myeloperoxidase (MPO) marker at day 8 and day 12 of GCSF-driven granulocyte unilineage culture post-nucleofection; (**E**) Morphological analysis of NegCTR mimic (**i**) and miR-34a-5p mimic-transfected cells (**ii**) after May–Grünwald–Giemsa staining at day 8 of GCSF-driven granulocyte differentiation post-nucleofection in a representative experiment. Thin arrows indicate myelocytes; thick arrows indicate band neutrophils. Magnification, ×1000. Abbreviations: EPO, erythropoietin; GCSF, granulocyte colony stimulating factor.

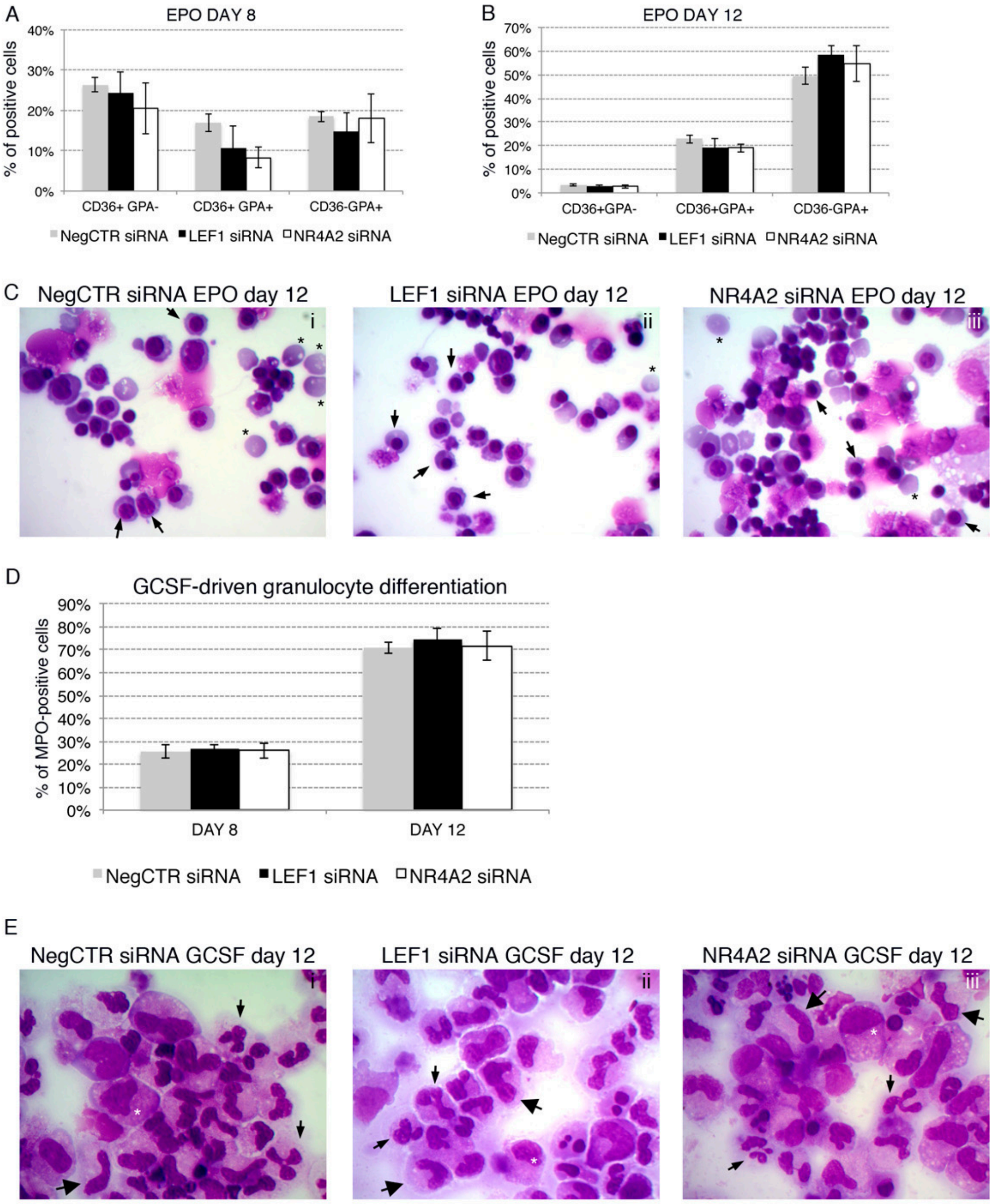


**Figure S3.** Effects of LEF1 and NR4A2 silencing on erythroid and granulocyte differentiation. (**A**,**B**) Flow cytometric analysis (mean ± SEM; $n = 3$) of CD36 and glycophorin A (GPA) expression at day 8 (**A**) and day 12 (**B**) of EPO-driven erythroid unilineage culture post-nucleofection; (**C**) Morphological analysis of NegCTR siRNA (**i**), LEF1 siRNA (**ii**) and NR4A2 siRNA-transfected cells (**iii**) after May–Grünwald–Giemsa staining at day 8 of EPO-induced erythroid differentiation post-nucleofection in a representative experiment. Arrows indicate erythroblasts; asterisks indicate reticulocytes that have already extruded the nucleus. Magnification, ×1000; (**D**) Flow cytometric detection (mean ± SEM; $n = 3$) of the myeloperoxidase (MPO) marker at day 8 and day 12 of GCSF-driven granulocyte unilineage culture post-nucleofection; (**E**) Morphological analysis of NegCTR siRNA (**i**), LEF1 siRNA (**ii**) and NR4A2 siRNA-transfected cells (**iii**) after May–Grünwald–Giemsa staining at day 8 of GCSF-driven granulocyte differentiation post-nucleofection in a representative experiment. Thick arrows indicate band neutrophils; thin arrows indicate two-lobed neutrophils or mature neutrophils; white asterisks indicate promyelocytes. Magnification, ×1000. Abbreviations: EPO, erythropoietin; GCSF, granulocyte colony stimulating factor.

**Table S1.** Ambion Silencer Select siRNA Sequences.

| Target Transcript | RefSeq ID | Sequence (Sense) | Targeted Exon(s) | Ambion Silencer Select siRNA Product |
|---|---|---|---|---|
| LEF1 | NM_001130713.2; NM_001130714.2; NM_001166119.1; NM_016269.4 | 5′-GUUGCUGAGUGUACUCUAAtt-3′ | Exons 7,8 | Silencer Select Pre-designed siRNA |
| NR4A2 | NM_006186.3 | 5′-GGCGAACCCUGACUAUCAAtt-3′ | Exon 6 | Silencer Select Validated siRNA |

**Table S2.** Differentially expressed genes between miR-34a-5p mimic and NegCTR mimic-transfected CD34+ cells.

| Probeset ID | Gene Symbol | Gene Title | RefSeq Transcript ID | Fold Change miR-34a-5p Mimic vs. NegCTR Mimic-Transfected CD34+ Cells | *p*-Value miR-34a-5p Mimic vs. NegCTR Mimic-Transfected CD34+ Cells |
|---|---|---|---|---|---|
| 11753714_a_at | *HBE1* | hemoglobin, epsilon 1 | NM_005330 | 4.17 | 0.0023 |
| 11755537_s_at | *HBG1 /// HBG2 /// LOC100653006 /// LOC100653319* | hemoglobin, γ A /// hemoglobin, γ G /// uncharacterized LOC100653006 /// unchar | NM_000184 /// NM_000559 /// XR_132577 /// XR_132954 | 3.47 | 0.0013 |
| 11744478_s_at | *HBG1 /// HBG2 /// LOC100653006 /// LOC100653319* | hemoglobin, γ A /// hemoglobin, γ G /// uncharacterized LOC100653006 /// unchar | NM_000184 /// NM_000559 /// XR_132577 /// XR_132954 | 3.44 | 0.0006 |
| 11754267_x_at | *HBA1 /// HBA2* | hemoglobin, α 1 /// hemoglobin, α 2 | NM_000517 /// NM_000558 | 3.40 | 0.0142 |
| 11715494_s_at | *HBG1 /// HBG2 /// LOC100653006 /// LOC100653319* | hemoglobin, γ A /// hemoglobin, γ G /// uncharacterized LOC100653006 /// unchar | NM_000184 /// NM_000559 /// XR_132577 /// XR_132954 | 3.31 | 0.0004 |
| 11729582_s_at | *CA1* | carbonic anhydrase I | NM_001128829 /// NM_001128830 /// NM_001128831 /// NM_001164830 /// NM_001738 | 3.06 | 0.0062 |
| 11758685_s_at | *CEACAM8* | carcinoembryonic antigen-related cell adhesion molecule 8 | NM_001816 | 2.97 | 0.0371 |
| 11753823_a_at | *S100A8* | S100 calcium binding protein A8 | NM_002964 | 2.85 | 0.0021 |
| 11758696_x_at | *HBG1 /// HBG2 /// LOC100653006 /// LOC100653319* | hemoglobin, γ A /// hemoglobin, γ G /// uncharacterized LOC100653006 /// unchar | NM_000184 /// NM_000559 /// XR_132577 /// XR_132954 | 2.77 | 0.0017 |
| 11720541_at | *LGALSL* | lectin, galactoside-binding-like | NM_014181 | 2.39 | 0.0111 |
| 11716523_at | *S100A9* | S100 calcium binding protein A9 | NM_002965 | 2.37 | 0.0002 |
| 11729583_x_at | *CA1* | carbonic anhydrase I | NM_001128829 /// NM_001128830 /// NM_001128831 /// NM_001164830 /// NM_001738 | 2.27 | 0.0297 |
| 11758545_s_at | *RHAG* | Rh-associated glycoprotein | NM_000324 | 2.09 | 0.0305 |
| 11725897_at | *TUBB1* | tubulin, beta 1 class VI | NM_030773 | 2.06 | 0.0468 |
| 11756809_a_at | *EFHC2* | EF-hand domain (C-terminal) containing 2 | NM_025184 | 2.06 | 0.0228 |
| 11758577_s_at | *ELOVL7* | ELOVL fatty acid elongase 7 | NM_001104558 /// NM_024930 | 2.06 | 0.0038 |
| 11720608_a_at | *S100B* | S100 calcium binding protein B | NM_006272 | 2.05 | 0.0261 |
| 11751647_a_at | *IL7R* | interleukin 7 receptor | NM_002185 | 2.01 | 0.0058 |
| 11725632_at | *NR4A2* | nuclear receptor subfamily 4, group A, member 2 | NM_006186 /// NM_173171 /// NM_173172 /// NM_173173 | −2.03 | 0.0245 |

**Table S2.** *Cont.*

| Probeset ID | Gene Symbol | Gene Title | RefSeq Transcript ID | Fold Change miR-34a-5p Mimic vs. NegCTR Mimic-Transfected CD34+ Cells | *p*-Value miR-34a-5p Mimic vs. NegCTR Mimic-Transfected CD34+ Cells |
|---|---|---|---|---|---|
| 11729641_a_at | *TPD52* | tumor protein D52 | NM_001025252 /// NM_001025253 /// NM_005079 | −2.06 | 0.0036 |
| 11715245_s_at | *IGLL1* | immunoglobulin lambda−like polypeptide 1 | NM_020070 /// NM_152855 | -2.15 | 0.0303 |
| 11718479_x_at | *STAR* | steroidogenic acute regulatory protein | NM_000349 /// NM_001007243 | −2.17 | 0.0057 |
| 11720051_at | *SPOCK1* | sparc/osteonectin, cwcv and kazal-like domains proteoglycan (testican) 1 | NM_004598 | −2.17 | 0.0183 |
| 11718477_a_at | *STAR* | steroidogenic acute regulatory protein | NM_000349 /// NM_001007243 | −2.24 | 0.0164 |
| 11726333_s_at | *LEF1* | lymphoid enhancer-binding factor 1 | NM_001130713 /// NM_001130714 /// NM_001166119 /// NM_016269 | −2.29 | 0.0042 |
| 11723339_at | *CTSG* | cathepsin G | NM_001911 | −2.29 | 0.0436 |
| 11745205_s_at | *TPD52* | tumor protein D52 | NM_001025252 /// NM_001025253 /// NM_005079 | -2.59 | 0.0027 |
| 11715306_s_at | *AREG /// AREGB* | amphiregulin /// amphiregulin B | NM_001657 /// XM_001125684 | −2.61 | 0.0045 |
| 11727965_at | *ELANE* | elastase, neutrophil expressed | NM_001972 | −2.65 | 0.0095 |
| 11754659_x_at | *TPD52* | tumor protein D52 | NM_001025252 /// NM_001025253 /// NM_005079 | −2.69 | 0.0079 |
| 11729643_s_at | *TPD52* | tumor protein D52 | NM_001025252 /// NM_001025253 /// NM_005079 | −2.89 | 0.0069 |
| 11756600_a_at | *TPD52* | tumor protein D52 | NM_001025252 /// NM_001025253 /// NM_005079 | −2.93 | 0.0047 |
| 11728026_x_at | *IGLL1* | immunoglobulin lambda-like polypeptide 1 | NM_020070 /// NM_152855 | −3.22 | 0.0157 |
| 11734171_at | *PRTN3* | proteinase 3 | NM_002777 | −3.81 | 0.0004 |
